# Evaluation of Antimicrobials Incorporated into Artificial Saliva: Analysis Against *Candida albicans*

**DOI:** 10.3390/jfb17020063

**Published:** 2026-01-27

**Authors:** Camila Alves Carneiro, Fenelon Martinho Lima Pontes, Karin Hermana Neppelenbroek, Rodrigo França, Vinicius Carvalho Porto

**Affiliations:** 1 Department of Prosthodontics and Periodontics, Dental School of Bauru, University of São Paulo, Bauru 17000-000, SP, Brazil; camila-alves@usp.br (C.A.C.);; 2 Dental Materials Research Lab, Department of Restorative Dentistry, Dr. Gerald Niznick College of Dentistry, Rady Faculty of Health Sciences, University of Manitoba, Winnipeg, MB R3E 0W2, Canada; 3 Department of Chemistry, Sao Paulo State University (UNESP), Bauru 17033-360, SP, Brazil

**Keywords:** artificial saliva, *Candida albicans*, nanoparticles, antimicrobials

## Abstract

Saliva is essential for maintaining oral health, and conditions like hyposalivation increase the risk of diseases. To address this, artificial saliva (AS) formulations incorporated with antimicrobials have been proposed. This study aimed to evaluate the antimicrobial activity and determine the minimum inhibitory concentration (MIC) and minimal fungicidal concentration (MFC) of AS formulations containing nystatin (Nys), chlorhexidine diacetate 98% (Chx), and silver nanoparticles (AgNp) against *Candida albicans* biofilm. The fungistatic and fungicidal properties of six groups (AS; AS + AgNp 2 mM; AS + AgNp 4 mM; AS + AgNp 6 mM; AS + Nys; AS + Chx) were assessed using the XTT colorimetric assay. Additionally, 35 denture base heat-polymerized acrylic resin specimens were prepared and treated with the antimicrobials, serving as substrates for *C. albicans* biofilm development over 3, 6, and 12 h. Biofilm growth was quantified by CFU/mL counting. All analyses were performed with a significance level of *p* < 0.05. Results demonstrated fungal load inhibition and a reduction in metabolic activity across all experimental groups (*p* < 0.05). Notably, AS + Nys, AS + Chx, and AS + AgNp 6 mM exhibited similar and significant inhibitory effects against *C. albicans* biofilm.

## 1. Introduction

Saliva is an oral fluid with several functions, among which are the cleaning and lubrication of soft and hard oral tissues, food bolus solubilization and formation, facilitation of taste perception, mastication, speech, retention of removable dentures, and the reduction in candidiasis activity in denture wearers [1]. Furthermore, the mechanics of salivary wetting are essential to create adhesion, cohesion, and surface tension, which ultimately lead to increased denture retention [2].

Thus, among the common problems affecting the elderly population, which significantly impact their quality of life, are salivary gland hypofunction, resulting in dry mouth (xerostomia) [3]. As the elderly live longer, coexisting with systemic diseases and consequently increasing their medication needs, the probability of developing problems related to hyposalivation increases proportionally, for this situation can be caused by radiation treatments for oral cancer, the presence of systemic conditions such as rheumatoid diseases, diabetes mellitus, Parkinson’s disease, and immune system dysfunctions, as well as various types of medications commonly used in the elderly population [4,5,6].

Hyposalivation can have a significant impact on daily life activities, mastication, reduced taste perception, dental caries, periodontal diseases, halitosis, difficulty in using dentures, and an increase in opportunistic infections. Xerostomia has been listed as one of the risk factors for denture stomatitis (DS) caused by *Candida* ssp. In addition, in xerostomic patients, the dryness of the oral mucosa makes it more susceptible to irritation and epithelial atrophy, leading to possible inflammation, fissuring, and ulceration [7,8].

One of the treatments of choice for these cases is salivary stimulants or substitutes containing thickening agents for longer relief and increased humidification and lubrication of oral surfaces, such as artificial saliva (AS), which has multiple components and is usually formulated as solutions, sprays, or gels [9]. Therefore, the main objectives of artificial saliva are to ensure lubrication of oral tissues, relieve dry mouth sensation, and protect dental tissues, possessing antimicrobial action to prevent the colonization of microorganisms such as *Candida* ssp. [10,11,12].

It is well known that the topical antifungal drug considered the gold standard for the treatment of DS is nystatin. However, several adverse effects have been reported regarding its use, depending on how it is administered. When applied topically (on the skin or mucous membranes), it can cause nausea and vomiting, while, when administered systemically (via oral or injectable routes), it can cause hepatotoxicity and gastrointestinal disorders [13]. Therefore, other antimicrobials have been researched and used against *C. albicans* biofilms, such as chlorhexidine, which is considered one of the best drugs in dentistry with high antimicrobial capacity and substantivity [14,15].

Another studied antimicrobial alternative are silver nanoparticles (AgNp), which are described in the literature for their antimicrobial potential across various biomedical and dental fields, as they have several properties closely related to the functionality, attractive physicochemical properties, specificity of antimicrobial action, and their biological behavior, including their high antifungal efficiency and relatively non-toxic, wide spectrum of bactericidal properties, anticancer properties, and other therapeutic abilities [16,17,18].

Considering that hyposalivation is one of the main risk factors for several oral diseases, such as DS, the development of an artificial saliva that possesses greater antimicrobial activity to reduce microorganism proliferation in xerostomic individuals can be an innovative alternative in the context of oral health, and may offer a beneficial preventive or supportive strategy, especially for patients undergoing radiotherapy who are removable denture wearers. Therefore, the aim of this study was to evaluate the antimicrobial activity and determine the minimum inhibitory concentration (MIC) and the minimal fungicidal concentration (MFC) of artificial saliva formulations incorporated with three different antimicrobials: nystatin (Nys), chlorhexidine diacetate 98% (Chx), and AgNp, against *Candida albicans* biofilm.

## 2. Materials and Methods

The antimicrobials selected for this study were powdered nystatin (Nys) (AC Ponta Grossa, Paraná, Brazil), 98% chlorhexidine diacetate (Chx) (Pharmácia Specífica Ltd. Bauru, São Paulo, Brazil) as a positive control, and different concentrations of silver nanoparticles (AgNp), each incorporated into the AS formulation, divided into 6 experimental groups (AS; AS + AgNp 2 mM; AS + AgNp 4 mM; AS + AgNp 6 mM; AS + Chx; AS + Nys) and a control group with no previous treatment.

### 2.1. Preparation of Artificial Saliva

The artificial saliva used in this study was developed by the Department of Biological Sciences—Biochemistry Discipline of the Bauru School of Dentistry. The composition is described in Table 1.

### 2.2. Synthesis and Characterization of Ag Nanoparticles

This step was performed at the Functional Nanostructured Materials Development Laboratory (UNESP/Bauru—Department of Chemistry). An aliquot of 20 mL of the AgNO_3_ solution (2, 4, and 6 mM) was placed in a jacketed quartz reactor connected to a thermostatic bath. The reactor was maintained shielded from ambient light, since Ag^+^ ions and silver nanoparticles are photosensitive and may undergo photoreduction, oxidation, or uncontrolled growth under illumination, which would compromise the reproducibility of the synthesis.

Then, 5 mL of the 10 mM sodium citrate solution was slowly added under constant magnetic stirring at room temperature (23 °C). After 2 min of stirring, the reaction mixture was irradiated with a 370 nm LED source (30 mW) for 30 min in the absence of external light to promote nanoparticle formation. After this period, the solution developed a dark color, indicating the formation of silver nanoparticles, which was later confirmed by UV–Vis spectroscopy.

The crystalline nature and phase identification of the Ag nanoparticles were analyzed using an X-ray diffractometer (Rigaku Miniflex 600, Rigaku Corporation, Tokyo, Japan) equipped with Cu Kα radiation (λ = 1.5406 Å), operating at 40 kV and 15 mA.

The formation of Ag nanoparticles was monitored using a UV–visible spectrophotometer (Agilent/HP 8453, Agilent Technologies, Santa Clara, CA, USA) over the wavelength range of 300–900 nm, employing a quartz cuvette with a 10 mm optical path length.

Transmission Electron Microscopy (TEM) analysis was performed to evaluate the morphology and size distribution of the Ag nanoparticles using a FEI Tecnai G2 F20 microscope (FEI Company, Hillsboro, OR, USA) operated at an accelerating voltage of 200 kV.

### 2.3. Manipulation and Standardization of the Inoculum

Frozen cells (−80 °C) of *C. albicans* (SC5314 strain) were seeded on plates containing Yeast Peptone Dextrose extract (YEPD—Difco^®^, Sparks, MD, USA) and incubated at 30 °C for 36 h in an incubator [19]. The suspension was then centrifuged at 4000 rpm for 5 min, and the cells were collected and washed with phosphate-buffered saline (PBS) and standardized to a concentration of 1 × 10^6^ cells/mL [20].

### 2.4. Determination of Minimum Inhibitory Concentration (MIC) and Minimum Fungicidal Concentration (MFC)

The minimum inhibitory concentration (MIC) was determined according to the EUCAST protocol (European Committee on Antimicrobial Susceptibility Testing) [21]. In 96-well plates (TPP^®^ 91015, Techno Plastic Products, Trasadingen, SH, Switzerland), containing Roswell Park Memorial Institute (RPMI-1640) culture medium supplemented with 2% glucose (Gibco®, Thermo Fisher Scientific, Waltham, MA, USA) serial dilutions of AgNp nanoparticles were prepared up to 10-fold. Each well received 100 µL of *C. albicans* suspension, and the plates were incubated at 35 °C. Control wells included predefined concentrations of chlorhexidine (Chx) at 4 mg/mL [20] and nystatin (Nys) at 32 mg/mL [19], as well as wells containing the inoculum plus RPMI-1640 solution. After 24 h, the XTT (2,3-bis(2-methoxy-4-nitro-5-sulfophenyl)-2H-tetrazolium-5-carboxanilide, Sigma Aldrich^®^ Inc., St. Louis, MO, USA) colorimetric assay was performed. A 0.5 mg/mL XTT solution and 0.1 mM menadione were prepared, and 100 µL aliquots from each well were transferred to new plates. Subsequently, 25 µL of the XTT solution was added to each well, and the plates were gently shaken. After incubating for 3 h at 37 °C, the plates were shaken again, and the absorbance was measured at 540 nm using a spectrophotometer (Biotek Synergy MX based Monochromator^®^, Winoosky, VT, USA) [19]. MIC was calculated by dividing the optical density (OD) of each concentration by the OD of the growth control, which was equivalent to 100% fungal growth, and these percentages were subtracted from 100. The data were then converted to a percentage of death [21]. The nanoparticles were considered to have antimicrobial activity if they caused an average reduction of ≥50%. After determining the concentration, all antimicrobials were incorporated into artificial saliva, and this procedure was repeated to determine the MIC of each solution [22]. The MFC was determined based on the previous test. From the concentrations of AS solutions with antimicrobials equal to or greater than 50%, and one concentration below, a 25 μL aliquot was taken, and these concentrations were inoculated on the surface of Sabouraud Agar in duplicate [22]. The plates were incubated at 37 °C, and after 24 h, Colony Forming Units (CFU/mL) were quantified, and the MFC was defined as the lowest concentration capable of causing fungal death. These assays were conducted in three independent experiments.

### 2.5. Preparation of Acrylic Resin Specimens

A total of 35 heat-polymerized denture base acrylic resin (Lucitone 550; Dentsply International Inc., Chicago, MI, USA) specimens were prepared (5 in each group) from a rectangular wooden matrix measuring 10 × 10 × 5 mm, with the help of a muffle. After deflasking the specimens, excess resin was removed using water sandpaper with grit 80 (Norton Abrasives) in a circular polisher (Struers-Panambra) at low speed, under refrigeration. Then, surface roughness was measured using a rugosimeter (Surftest SJ-301; Mitutoyo Corporation, Kawasaki, Kanagawa, Japan), with mean surface roughness of 0.3 μm (Ra). After that, the samples were sterilized [23,24].

### 2.6. Surface Treatment and C. albicans Biofilm Development

In 50 mL Falcon tubes, the predefined concentrations of antimicrobials were mixed with the artificial saliva formulation according to the MIC values found. These mixtures were homogenized using a glass beaker homogenizer at room temperature (25 ± 2 °C) for 15 min, left for 30 min, filtered through Whatman filter paper No. 1, and stored in an airtight container for the subsequent experiment [25]. The specimens were immersed for 60 min at 37 °C in the artificial saliva formulations [26]. Then, they were immersed in 1 mL of the previously standardized cell suspension and incubated for 90 min at 37 °C, at 75 rpm, in an incubator with orbital agitation (Shaker—Model 430/RDPB, Nova Ética^®^, Scientific Products and Equipment, Vargem Grande do Sul, SP, Brazil). Afterwards, the specimens were washed in 1 mL of PBS to remove non-adherent organisms and subsequently immersed in 1 mL of RPMI-1640 solution for 3, 6, and 12 h at 37 °C, at 75 rpm [23,27].

### 2.7. Quantification of C. albicans Biofilm by Colony Forming Units per Milliliter (CFU/mL)

Following each evaluation period, the specimens were carefully rinsed with 1 mL of PBS. The biofilm was then detached from the surface using a cell scraper (Costar^®^ 3010; Corning Inc., Corning, NY, USA) and preserved in 1 mL of PBS. These suspensions underwent serial dilution (10^−1^ to 10^−4^), and aliquots of 50 µL from the selected dilution (10^−3^) were plated in triplicate on Sabouraud Dextrose Agar (Accumedia Manufacturers, Lansing, MI, USA). The plates were incubated at 37 °C for 24 h. After incubation, the colonies were counted, and the results were reported as average CFU/mL values [19].

### 2.8. Statistical Analyses

Data normality was assessed using the Shapiro–Wilk test (*p* > 0.05). Paired *t*-tests were used to compare the antifungal activity of each AgNp concentration before and after incorporation into AS. CFU data were analyzed using a two-way ANOVA, followed by Tukey’s post hoc test (*p* < 0.05). The primary outcome for the MIC/MFC assays was the percentage reduction in metabolic activity. For the CFU analysis, the primary outcome was the mean CFU/mL. The CFU assay was designed as a pilot experiment; the sample size (n = 5) was based on a previous in vitro study [19].

## 3. Results

### 3.1. Transmission Electron Microscopy (TEM) of Ag Nanoparticles

**Figure 1:**
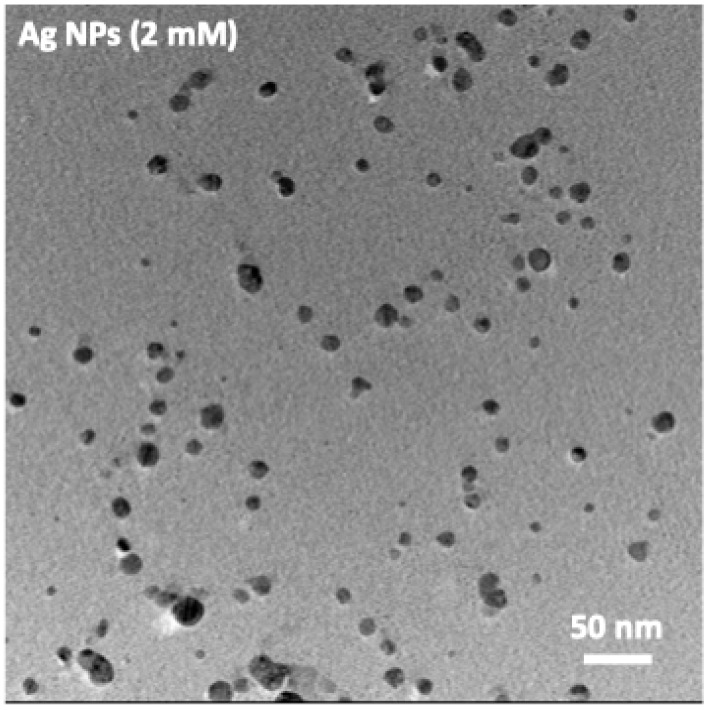
TEM micrographs of Ag nanoparticles synthesized at 2 mM AgNO_3_.

The TEM micrographs of the Ag nanoparticles synthesized at 2 mM AgNO_3_ show a markedly more uniform colloidal population compared to the higher-concentration samples, Figure 1. The particles are predominantly spherical or quasi-spherical and display a relatively narrow size distribution, with no evident formation of faceted or irregular morphologies. This morphological uniformity is fully consistent with the UV–Vis spectrum of the 2 mM sample, which exhibited a narrow and symmetric LSPR band centered at 408 nm, characteristic of small and nearly monodisperse spherical Ag nanoparticles.

**Figure 2:**
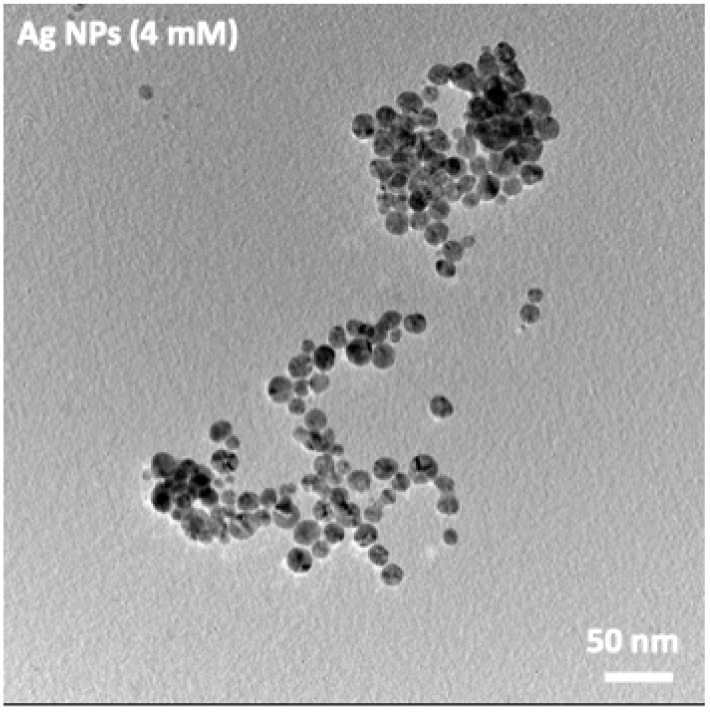
TEM micrographs of Ag nanoparticles synthesized at 4 mM AgNO_3_.

The TEM images of the Ag nanoparticles synthesized at 4 mM AgNO_3_ reveal an increase in particle size relative to the 2 mM sample, accompanied by the onset of morphological heterogeneity (Figure 2). Although spherical particles remain predominant, a fraction of larger crystallites with slightly faceted or irregular outlines can be observed, indicating the transition from a nearly monodisperse regime to a broader size distribution. These features corroborate the UV–Vis results, which showed a broader LSPR band (FWHM ≈ 75 nm) and a red-shift to 423 nm, both consistent with an increase in average particle size and polydispersity at intermediate precursor concentration.

**Figure 3:**
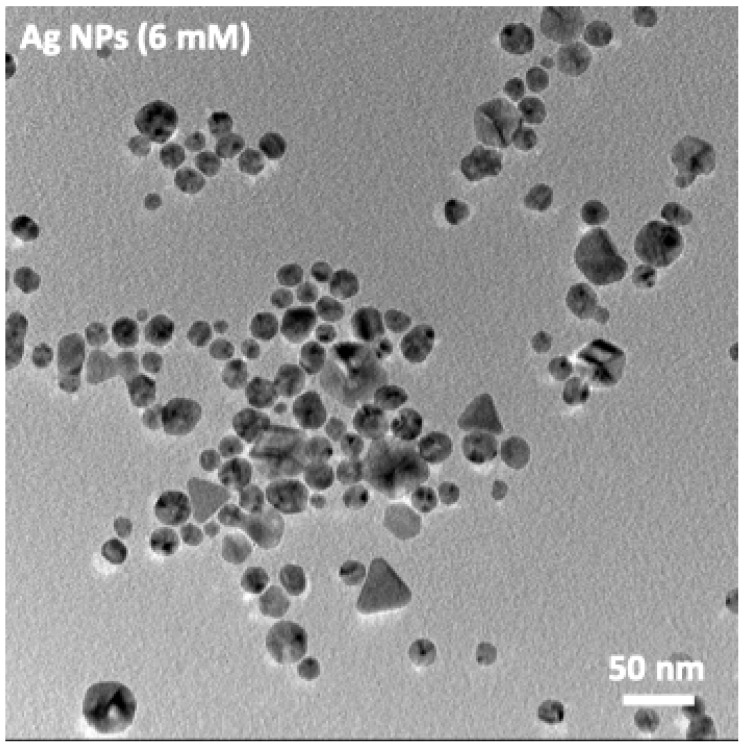
TEM micrographs of Ag nanoparticles synthesized at 6 mM AgNO_3_.

In contrast, at 6 mM AgNO_3_, a marked increase in both particle size and morphological diversity is observed. The TEM micrographs reveal a polymorphic structure, presenting a highly polydisperse population of nanoparticles with sizes ranging from a few nanometers up to several tens of nanometers. The particles display predominantly quasi-spherical morphologies, although a noticeable fraction of faceted and irregularly shaped crystallites is also observed. This broader size and shape distribution is fully consistent with the UV–Vis results (Figure 3).

### 3.2. Minimum Inhibitory Concentration (MIC) and Minimum Fungicidal Concentration (MFC)

**Figure 4:**
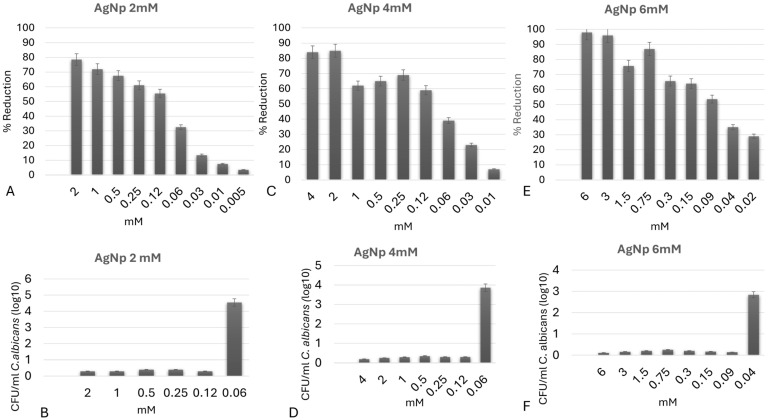
MIC (**A**,**C**,**E**) and MFC (**B**,**D**,**F**) of silver nanoparticles (AgNp) at concentrations of 2 mM, 4 mM, and 6 mM against planktonic *Candida albicans.* (**A**,**B**) AgNp 2 mM; (**C**,**D**) AgNp 4 mM; (**E**,**F**) AgNp 6 mM. Error bars represent standard deviation of three independent experiments performed in triplicate.

Firstly, the MIC of AgNp suspensions at the three initial experimental concentrations was evaluated, and all demonstrated fungistatic and fungicidal activity against *C. albicans* planktonic cells at different levels. After 24 h of incubation (MIC and MFC), the silver nanoparticles at initial concentration of 6 mM resulted in a reduction of over 50% in fungal metabolic activity and 100% inhibition in colony formation (CFU/mL) at a final concentration of 0.09 mM (~9.7 µg/mL Ag), and the fungistatic and fungicidal potential of the nanoparticles with initial concentrations of 2 mM and 4 mM was observed at final concentrations of 0.12 mM (~12.9 µg/mL Ag) (Figure 4A–F).

**Figure 5:**
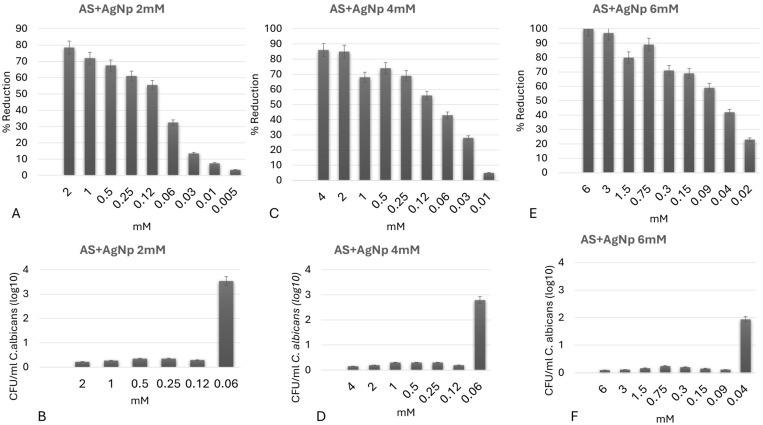
MIC (**A**,**C**,**E**) and MFC (**B**,**D**,**F**) of silver nanoparticles (AgNp) incorporated into artificial saliva (AS) at concentrations of 2 mM, 4 mM, and 6 mM against planktonic *Candida albicans*. (**A**,**B**) AS + AgNp 2 mM; (**C**,**D**) AS + AgNp 4 mM; (**E**,**F**) AS + AgNp 6 mM. Error bars represent standard deviation of three independent experiments performed in triplicate.

After the initial analysis, the AgNp suspensions were incorporated into AS, and the influence of AS on the antifungal activity of the AgNp was evaluated. After 24 h of incubation (MIC and MFC), the AgNp suspensions incorporated into AS, regardless of the initial concentration, demonstrated the same fungistatic and fungicidal activity against *C. albicans* planktonic cells as observed in the previous test. The incorporation of AgNp into AS showed a slight enhancement of the antifungal effect, but without significant differences between them (*p* = 0.203), maintaining the same final concentration (Figure 5A–F).

**Figure 6:**
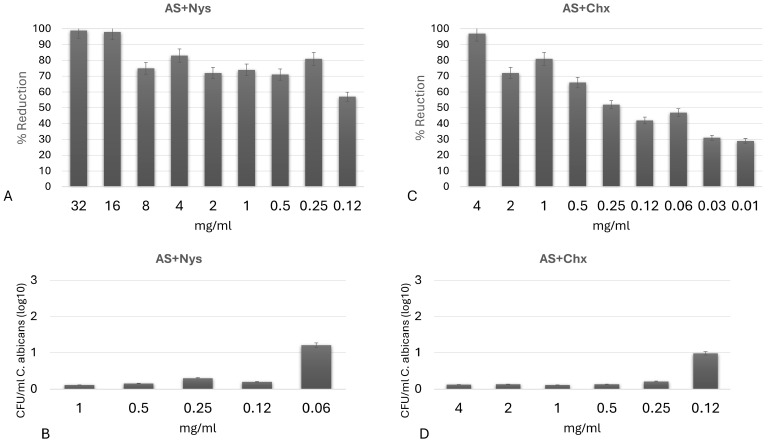
MIC (**A**,**C**) and MFC (**B**,**D**) of artificial saliva (AS) formulations containing nystatin (Nys) and chlorhexidine diacetate 98% (Chx) against planktonic *Candida albicans*. (**A**,**B**) AS + Nys; (**C**,**D**) AS + Chx. Error bars represent standard deviation of three independent experiments performed in triplicate.

As expected, Nys incorporated into artificial saliva reduced fungal metabolic activity by more than 50% at all concentrations tested, with the minimum concentration of 0.12 mg/mL. Chx incorporated into AS exhibited antifungal potential starting at a concentration of 0.25 mg/mL. When incorporated into AS, both Nys and Chx showed 100% inhibition of *C. albicans* biofilm at concentrations equal to or higher than those determined in the previous test (Figure 6A–D).

### 3.3. Quantitative Analysis of C. albicans Biofilm (CFU/mL)

The data from the CFU assay are presented in Table 2. The groups AS + Chx, AS + Nis, AS + AgNp 4 mM, and AS + AgNp 6 mM exhibited high inhibitory values at all evaluated time points, showing statistically significant differences when compared to the AS and control group (*p* < 0.05). At 3, 6, and 12 h, it was evident that the AS + AgNp 2 mM group showed statistical differences from the control group, but statistically differed from the AS group at 3 and 12 h of evaluation. The AS group showed results similar to the control group when evaluated at 12 h. However, despite not demonstrating high inhibitory power, the growth of the *C. albicans* biofilm at all evaluation times showed a statistical difference compared to the control group (*p* < 0.05) when evaluated at 3 and 6 h.

## 4. Discussion

In this study, the efficacy of artificial saliva incorporated with antimicrobials was investigated in order to enhance its antimicrobial activity, specifically against *C. albicans*, the main agent responsible for DS in denture wearers [28], since artificial saliva is an important therapeutic tool for patients with hyposalivation or xerostomia, and dry mouth might increase the risk of opportunistic infections [29].

Silver nanoparticles were chosen due to several advantages associated with these compounds, such as optimized pharmacological activity, increased bioavailability, loading of therapeutic substances, and reduction in side effects caused by drugs [30]. In this study, silver nanoparticles at all evaluated concentrations showed high antifungal potential, which corroborates the results obtained by Monteiro et al. [31], who evaluated the effect of silver nanoparticles against *C. albicans* and *Candida glabrata* adhered cells and biofilms, obtaining results with high inhibition power. This is due to the fact that AgNPs affect intracellular metabolic processes, causing severe morphological changes in the fungal cells manifested by disruption of the cell membrane structure, resulting in fungal cell death. Minor non-linear fluctuations across adjacent concentrations, such as occasional higher inhibition at an intermediate dose, may result from biological variability inherent to nanoparticle–cell interactions and have also been described in prior antifungal studies [19,32,33,34,35].

Incorporation into artificial saliva, metal nanoparticles are not a common material of choice among researchers, as there are reports in the literature of antimicrobial activity of natural plant compounds incorporated into AS, such as the work of Manosroi et al. [25], with Ceylon spinach against *Streptoccocus mutans*. However, in an in vitro study, Lawaoska et al. [36] incorporated core–shell magnetic gold-coated nanoparticles into AS, and obtained inhibition results greater than 50% against *S. mutans* and *C. albicans* biofilms, once nanoparticles release toxic metals that react with macromolecules, impairing their function and inhibiting microbial growth or causing cell death, and generating reactive oxygen species (ROS) that damage DNA, RNA, and proteins of microbial cells [37].

Despite existing studies exploring AgNP interactions with human saliva, the authors have not yet found any research using AgNPs incorporated into artificial saliva for study its antifungal capacity, and in this study, when incorporated into AS, silver nanoparticles, at concentrations of 2 mM, 4 mM, and 6 mM, not only maintained their fungistatic and fungicidal capacity, but also showed slight potentiation, indicating that the presence of the chemical agents in the artificial saliva improved the antimicrobial effect of the AgNPs, suggesting an affinity of AgNPs to these agents, even with the carboximethylcellulose (CMC), present in the base formulation of the AS used in this study, as previous studies [38,39] showed that the presence of CMC inhibited proteins such as hemoglobin and cystatin when incorporated into artificial saliva.

In fact, based on the MIC assay, it was observed that AgNPs incorporated with AS showed both fungistatic and fungicidal activity against planktonic *C. albicans* cells in a concentration-dependent manner. In other words, as the concentration of the NPs increased, there was a greater reduction in fungal metabolic activity and inhibition of colony formation. Although the three concentrations remained statistically distinct from one another after incorporation, the AS itself did not potentiate or suppress the intrinsic activity of each AgNP concentration. This superior performance is attributed to the physicochemical features of the 6 mM AgNPs, whose broader size distribution, larger particle domains, and increased surface reactivity enhance their interaction with fungal cells and disrupt biofilm formation more effectively [40,41].

Overall, the results demonstrate a clear structure–property–function relationship: the controlled tuning of nanoparticle size and polydispersity through precursor concentration directly influences their optical properties and biological efficacy. Although all AgNp concentrations exhibited antifungal activity, the 2 mM suspension showed reduced performance at 6–12 h when compared to 4 mM and 6 mM. This trend has been reported in previous studies evaluating nanoparticle-based antimicrobials, in which lower concentrations often show faster loss of activity over time due to reduced availability of active silver species or less stable nanoparticle suspensions [42,43].

In the present study, the AS + AgNp 6 mM group showed the highest inhibition among the 3 concentrations of AgNp evaluated, approaching the inhibition levels observed using nystatin and chlorhexidine. Nystatin, considered the gold standard against *C. albicans* biofilms, is a polyene antifungal that acts on ergosterol in fungal cell membranes, increasing membrane permeability and leading to cell death [44]. In this study, the incorporation of nystatin into artificial saliva did not alter its antifungal properties; in fact, it inhibited fungal biofilm growth at all evaluated concentrations, these results were supported by Salehi et al. [45], who evaluated nystatin and chlorhexidine against two *C. albicans* strains, one resistant to fluconazole and one susceptible, and observed that both showed antifungal potential, especially when combined, which is in contrast with Scheibler et al. [46], who stated that according to MIC results in their study, nystatin’s efficacy was more affected than chlorhexidine’s when used in combination, and that the bioavailability of nystatin is reduced by saliva dilution.

The results obtained in this study with chlorhexidine also demonstrated that this antimicrobial has high levels of fungal inhibition, which aligns with Ellepola and Samaranayake [47], who stated that, even at low concentrations, chlorhexidine digluconate causes rupture in the yeast cell membrane, making it a potent antifungal. And Scheibler et al. [15], who evaluated the susceptibility of *C. albicans* biofilms against chlorhexidine and nystatin, and both combined, found a MIC value of chlorhexidine of 5.1 μg/mL in suspended artificial saliva solutions. Additionally, chlorhexidine has high substantivity, a characteristic associated with its potential as an antimicrobial, as it is released gradually, promoting its efficacy for a long period [46].

The CFU assay in this study is an experimental test conducted on heat-polymerized PMMA acrylic resin, commonly used for complete denture bases, and demonstrated that AS formulations containing AgNp, Chx, or Nys effectively reduced early-stage biofilm formation. This is clinically relevant given that heat-polymerization increases porosity and may facilitate microbial adhesion [48,49]. Since complete denture wearers are susceptible to *C. albicans* infection, a susceptibility that is potentiated by the absence of saliva [50]. Previous studies have evaluated the effect of AS on fungal biofilm adhesion on acrylic resin, such as Oncul et al. [51], who evaluated two types of artificial saliva in *C. albicans* adhesion on heat-polymerized acrylic resin and observed significant differences with Biotene Oral Balance Gel artificial saliva, which contains lysozyme, lactoferrin, and peroxidase in fungal inhibition.

AgNPs incorporated into AS demonstrated inhibition power in the three periods evaluated (3 h, 6 h, and 12 h), especially the AS + AgNp 6 mM and AS + AgNp 4 mM groups. Results that align with several authors, such as Peralta et al. [19], who evaluated the incorporation of AgNPs into denture adhesive, in the adhesion of *C. albicans* biofilms on acrylic resin, and obtained inhibition results for up to 12 h. In addition, Li et al. [52] assessed the effect of denture base resin containing silver nanoparticles on *C. albicans* adhesion and biofilm formation, concluding an antifungal activity and an inhibitory effect on adhesion and biofilm formation by the AgNPs.

As expected, both chlorhexidine and nystatin showed high antifungal potential in all periods evaluated. The findings are consistent with Redding et al. [53], who evaluated chlorhexidine diacetate incorporated thin-film polymer on acrylic resin specimens after 24 h of incubation, compared to other antimicrobials like nystatin, and although all antifungals promoted satisfactory biofilm inhibition, the chlorhexidine diacetate group showed significantly better results. The findings of this study are also supported by Venante et al. [20], who evaluated chlorhexidine incorporated with fibrin biopolymer in heat-polymerized acrylic resin, and found that it significantly reduced *C. albicans* biofilm formation and growth in all of the evaluated assays and periods, and the study conducted by Maluf et al. [54], who evaluated the physical properties and antifungal activities of acrylic resins after the incorporation of chlorhexidine diacetate salt against *C. albicans* biofilm.

The effect of AS without antimicrobials on acrylic resin was also evaluated, and although it did not show inhibition power compared to the groups with antimicrobials, it showed slight antifungal potential. The AS used in this study basically contains inorganic components, such as the thickening agent CMC. In this regard, a study conducted by Edgerton et al. [55] stated that mucins present in human saliva may play a role in *C. albicans* adhesion on acrylic resin. However, previous studies that evaluated the effect of artificial saliva on *C. albicans* adhesion in heat-polymerized acrylic have been reported. Paranhos et al. [56], compared commercial AS combined with denture adhesive, soap, and only the adhesive, on fungal biofilm adhesion in complete dentures and concluded that the use of Corega (denture adhesive) combined with Oral Balance (artificial saliva) was the most effective method for reducing biofilm levels.

However, Oncul et al. [51] evaluated two types of commercial artificial saliva, and both artificial saliva showed higher *C. albicans* adhesion than distilled water. It should also be noted that the artificial saliva formulation contains methylparaben and propylparaben preservatives with documented antifungal activity against *Candida* spp. [57]. Their presence may help explain the mild inhibitory effect observed for the AS group at early time points.

It is also important to remind that Amphotericin B (AmB) remains a cornerstone for the treatment of deep and invasive mycoses owing to its broad-spectrum fungicidal activity, low resistance rates, and enduring clinical relevance even in the era of newer antifungals [58,59,60,61,62,63,64,65,66,67,68,69]. Nevertheless, its clinical deployment is constrained by a narrow therapeutic index, infusion-related reactions, and the need for slow intravenous administration with close monitoring [59,61]. These limitations are magnified by intrinsically low aqueous solubility (<0.001 mg/mL), which underlies poor oral bioavailability and off-target interactions that contribute to nephrotoxicity and electrolyte derangements (e.g., hypokalemia, hypomagnesemia) [60,61,64]. While lipid formulations mitigate toxicity and improve tolerability, comparative trials and meta-analyses indicate that echinocandins frequently match or surpass AmB for candidemia with fewer adverse events, supporting echinocandins as first-line in many adult scenarios [62,70,71,72,73,74]. Population- and context-specific nuances are critical: in premature infants and patients with renal dysfunction, lipid-based AmB (e.g., liposomal AmB, ABCD) can be effective and better tolerated than deoxycholate formulations [61,75,76]; fluconazole can be superior in certain non-neutropenic, low-risk candidemia settings [66]; and Candida auris presents variable—and often weak—AmB activity across clades, emphasizing the importance of early and effective killing to avoid cardiac and CNS complications [58].

To address delivery and toxicity constraints, several strategies are advancing. Oral and targeted delivery platforms—cochleates, nanoparticles, and nano/micro-carriers—aim to increase solubility/bioavailability, localize exposure, and reduce nephrotoxicity, with encouraging preclinical and translational signals [59,60,72,77]. Liposomal AmB demonstrates enhanced activity against Candida biofilms and utility in device-associated infections (e.g., catheter lock therapy), aligning with its improved therapeutic index in clinical practice [67,68,76]. Combination therapy is a complementary approach: echinocandins plus AmB show synergistic killing in vitro and in invertebrate models of *C. auris*, potentially reducing required AmB doses [70,71,78,79,80,81]; azoles such as isavuconazole combined with liposomal AmB have achieved clinical success in refractory invasive candidiasis (case-level evidence) [81]; and non-antifungal adjuncts (e.g., HIV protease inhibitors, nicotinamide, lansoprazole) can potentiate AmB activity in vitro or animal models, suggesting repurposing pathways [59,81]. 

In neonates, randomized data show micafungin can be as effective or superior to AmB with fewer adverse effects [64], and adult RCTs further support micafungin’s non-inferiority to liposomal AmB alongside better tolerability [74]. Collectively, these findings support a contemporary paradigm: retain AmB as a key agent for severe or resistant infections while leveraging lipid/nano-formulations and rational combinations to expand its therapeutic index and tailor therapy to pathogen, host, and infection site [63,67,69]. Given AmB’s parenteral administration requirements, narrow therapeutic index, and intrinsically poor aqueous solubility, it is not well-suited for incorporation into artificial saliva or topical in vitro protocols targeting denture-associated biofilms. Therefore, we focused on agents with established topical applicability and favorable safety profiles in oral environments—nystatin, chlorhexidine, and silver nanoparticles. This alignment ensures clinical relevance for xerostomic and denture-wearing patients, consistent with the intended preventive and supportive use of artificial saliva.

This study has some limitations that should be considered. First, the antifungal activity was assessed using a short-term biofilm model (3–12 h). Although these periods do not represent mature or multispecies denture biofilms, they were intentionally selected to simulate the clinical routine of xerostomic denture wearers, who typically remove dentures for hygiene and reapply artificial saliva within similar intervals. Future studies should include longer incubation periods and mixed-species biofilms to better approximate clinical complexity. Second, while the artificial saliva formulation followed a standardized composition, no preliminary physicochemical assessments were performed prior to its experimental application.

## 5. Conclusions

Based on the results of this study, artificial saliva supplemented with silver nanoparticles, chlorhexidine, and nystatin demonstrated significant antifungal activity against *Candida albicans* in both planktonic and early biofilm phases. Among the experimental formulations, AS + Nys, AS + Chx, and AS + AgNp 6mM exhibited the highest levels of inhibition, with the AgNp 6 mM group showing an antifungal effect that approached those of the conventional antifungal agents. These findings indicate that artificial saliva can serve as a potential delivery vehicle for antifungal agents, for enhancing early-stage biofilm control in xerostomic denture wearers.

Further studies involving extended biofilm maturation periods, cytotoxicity, clinical isolations, and biocompatibility assessments are necessary to support the translational potential of these formulations.

## Figures and Tables

**Table 1:** Formulation of artificial saliva.

Component	Concentration	Component	Concentration
Sodium bicarbonate	0.21%	Dipotassium phosphate	0.12%
Calcium Chloride	0.06%	Magnesium Chloride	0.012%
Potassium chloride	0.08%	Methylparaben	0.01%
Propylparaben	0.01%	Carboxymethylcellulose	0.8%

**Table 2:** Mean CFU/mL values (×10^3^) ± standard deviation.

Group	3 h	6 h	12 h
CT	2.332 ± 0.282 Aa	2.764 ± 0.282 Ba	2.883 ± 0.282 Ba
AS	2.029 ± 0.396 Ab	2.156 ± 0.396 Bb	2.868 ± 0.396 Ca
AS + Chx	0.0 Ac	0.0 Ac	0.0 Ab
AS + Nys	0.0 ± 0 Ac	0.0 ± 0 Ac	0.7 ± 0.1 Ab
AS + AgNp 2 mM	0.0 Ac	2.344 ± 0.123 Bb	2.170 ± 0.123 Cc
AS + AgNp 4 mM	0.0 Ac	0.0 Ac	1.176 ± 0.3 Ab
AS + AgNp 6 mM	0.0 Ac	0.0 Ac	0.0 Ab

CT: Control Group; AS: Artificial Saliva; AgNp: Silver Nanoparticles. Horizontally, different uppercase letters indicate statistical differences between experimental periods for the same group (*p* < 0.05). Vertically, different lowercase letters indicate statistical differences between groups within the same period (*p* < 0.05).

## Data Availability

The original contributions presented in the study are included in the article, further inquiries can be directed to the corresponding author.
